# Incidence and impact of acute kidney injury on patients with implantable left ventricular assist devices: a Meta-analysis

**DOI:** 10.1080/0886022X.2020.1768116

**Published:** 2020-05-20

**Authors:** Charat Thongprayoon, Ploypin Lertjitbanjong, Wisit Cheungpasitporn, Panupong Hansrivijit, Tibor Fülöp, Karthik Kovvuru, Swetha R. Kanduri, Paul W. Davis, Saraschandra Vallabhajosyula, Tarun Bathini, Kanramon Watthanasuntorn, Narut Prasitlumkum, Ronpichai Chokesuwattanaskul, Supawat Ratanapo, Michael A. Mao, Kianoush Kashani

**Affiliations:** a Division of Nephrology and Hypertension, Mayo Clinic, Rochester, MN, USA; b Department of Internal Medicine, Bassett Medical Center, Cooperstown, NY, USA; c Division of Nephrology, University of Mississippi Medical Center, Jackson, MS, USA; d Department of Internal Medicine, University of Pittsburgh Medical Center Pinnacle, Harrisburg, PA, USA; e Department of Medicine, Division of Nephrology, Medical University of South Carolina, Charleston, SC, USA; f Medicine Service, Ralph H. Johnson VA Medical Center, Charleston, SC, USA; g Department of Cardiovascular Medicine, Mayo Clinic, Rochester, MN, USA; h Department of Internal Medicine, University of Arizona, Tucson, Arizona, USA; i Department of Medicine, University of Hawaii, Honolulu, HI, USA; j Faculty of Medicine, King Chulalongkorn Memorial Hospital, Chulalongkorn University, Bangkok, Thailand; k Division of Cardiology, Department of Medicine, Phramongkutklao College of Medicine, Bangkok, Thailand; l Division of Nephrology and Hypertension, Mayo Clinic Health System, Jacksonville, FL, USA; m Division of Pulmonary and Critical Care Medicine, Department of Medicine, Mayo Clinic, Rochester, MN, USA

**Keywords:** Acute kidney injury, AKI, left ventricular assist device, LVAD, epidemiology, meta-analysis

## Abstract

**Background:**

We aimed to evaluate the acute kidney injury (AKI) incidence and its associated risk of mortality in patients with implantable left ventricular assist devices (LVAD).

**Methods:**

A systematic literature search in Ovid MEDLINE, EMBASE, and Cochrane Databases was conducted through January 2020 to identify studies that provided data on the AKI incidence and AKI-associated mortality risk in adult patients with implantable LVADs. Pooled effect estimates were examined using random-effects, generic inverse variance method of DerSimonian-Laird.

**Results:**

Fifty-six cohort studies with 63,663 LVAD patients were enrolled in this meta-analysis. The pooled incidence of reported AKI was 24.9% (95%CI: 20.1%–30.4%) but rose to 36.9% (95%CI: 31.1%–43.1%) when applying the standard definition of AKI per RIFLE, AKIN, and KDIGO criteria. The pooled incidence of severe AKI requiring renal replacement therapy (RRT) was 12.6% (95%CI: 10.5%–15.0%). AKI incidence did not differ significantly between types of LVAD (*p* = .35) or indication for LVAD use (*p* = .62). While meta-regression analysis did not demonstrate a significant association between study year and overall AKI incidence (*p* = .55), the study year was negatively correlated with the incidence of severe AKI requiring RRT (slope = −0.068, *p* < .001). The pooled odds ratios (ORs) of mortality at 30 days and one year in AKI patients were 3.66 (95% CI, 2.00–6.70) and 2.22 (95% CI, 1.62–3.04), respectively. The pooled ORs of mortality at 30 days and one year in severe AKI patients requiring RRT were 7.52 (95% CI, 4.58–12.33) and 5.41 (95% CI, 3.63–8.06), respectively.

**Conclusion:**

We found that more than one-third of LVAD patients develop AKI based on standard definitions, and 13% develop severe AKI requiring RRT. There has been a potential improvement in the incidence of severe AKI requiring RRT for LVAD patients. AKI in LVAD patients was associated with increased 30-day and 1 year mortality.

## Introduction

Implantable left ventricular assist devices (LVADs) are increasingly utilized as a bridge to heart transplantation or destination therapy for patients with end-stage heart failure [1–7]. The use of LVADs is shown to be associated with reduced mortality in patients on heart transplantation waiting lists, and they improve quality of life and functional status in advanced heart failure patients [8]. LVADs alleviate the cardiovascular load on a failing heart and have shown notable advantages in treating patients with advanced heart failure, providing prolonged survival and improvement in the quality of life [9,10]. Clinical outcomes after LVAD implantation have significantly improved over the past decade, with 1 year and 2 year survival of 83% and 73%, respectively [11, 12]. In the United States, the number of LVAD implantations rose, from only 459 implants in 2008 to a total of 2,118 implants in 2017 [11].

Despite the LVAD benefits mentioned above, several studies have reported persistent adverse complications following LVAD implantation, such as bleeding, cardiac arrhythmias, hypertension, sepsis, disabling stroke, and acute kidney injury (AKI) [8,13]. Post-implantation AKI has been associated with negative impacts on patient outcomes, including right ventricular failure, arrhythmia, and reduced survival [14,15]. The reported AKI incidence among LAVD patients widely ranged from 4–70%. This variability is likely due to the use of non-standardized AKI definitions in previous studies [15–70]. Furthermore, the mortality associated with AKI and current trends of AKI occurrence in LVAD patients are unclear [18,21,22,24,26,40,44,46,52,54,57,63,65].

This systematic review and meta-analysis were conducted to summarize the AKI incidence and mortality risk among adult patients with LVADs.

## Methods

The protocol for this meta-analysis is registered with PROSPERO (no. CRD42020134592). The meta-analyses were conducted in adherence to the Preferred Reporting Items for Systematic Reviews and Meta-Analysis (PRISMA) statement [71].

### Search strategy

Two investigators (CT and PL) independently searched for published clinical trials or observational studies indexed in MEDLINE, EMBASE and the Cochrane databases from inception to January 2020 using a search strategy (S1 in online Supplementary Data 1) that included the terms “left ventricular assist device”, “LVAD”, “ventricular assist device”, “acute kidney failure”, “acute kidney injury” and “renal replacement therapy”. No language restrictions were applied in this systemic review and meta-analysis. A manual search for additional pertinent studies and review articles using references from the retrieved articles was also completed.

### Study eligibility criteria

Two main criteria were used for study inclusion. First, the study had to report the incidence of AKI or severe AKI requiring renal replacement therapy (RRT), and AKI associated mortality risk in adult patients with LVADs aged at least 18 years. Second, the study had to include data assessing AKI incidence or mortality risk with 95% confidence intervals (CIs) (or sufficient raw data for the calculation). Patients were excluded if they only used a temporary, short-term, non-implantable LVAD during a hospitalization. Study eligibility was independently determined by two investigators (CT and PL). Differences were resolved by mutual consensus.

A standardized data collection form was used to obtain the following information from each study: title, name of the first author, year of study, year of publication, country of origin, number of participants, demographic data of participants, the method used to diagnose the outcomes of interest (AKI incidence and associated mortality), the average duration of follow-up, adjusted and unadjusted risk ratios and their corresponding 95% CI, and list of confounders that were adjusted for in the multivariate analyses. To ensure accuracy, both investigators independently performed this data extraction process. Any data discrepancy was resolved by referring back to the original articles. The Newcastle-Ottawa quality assessment scale was utilized to appraise the quality of observational studies [72].

### Statistical analysis

The meta-analysis of combined data was performed using a random-effects, generic inverse variance method of DerSimonian and Laird [73]. We assessed the overall incidence of AKI, which was defined by the consensus definitions provided by the Risk, Injury, Failure, Loss of kidney function, and End-stage kidney disease (RIFLE) [74], Acute Kidney Injury Network (AKIN) [75], and Kidney Disease: Improving Global Outcomes) (KDIGO) [76] classifications. We did not impute missing values for any outcomes in our analyses. A random-effect model was used to pool AKI incidence and AKI-associated mortality risk due to the possibility of between-study variance. Heterogeneity among included studies was statistically evaluated by the Cochrane’s Q test and the *I*^2^ statistic. Heterogeneity was considered insignificant when *I*^2^ of ≤25%, low when *I*^2^ of 26–50%, moderate when *I*^2^ of 51–75%, and high when *I*^2^ of ≥75% [77]. Per Cochrane, publication bias was assessed using a funnel plot. Funnel plot asymmetry was further confirmed with Egger’s test if there were >10 available studies [78]. All analysis was performed using The Comprehensive Meta-Analysis software version 3.3.070 (Biostat Inc, New Jersey, USA). The data underlying the results presented in the study are available through the Open Science Framework (https://osf.io/8hk35/)

## Results

**Figure 1.**
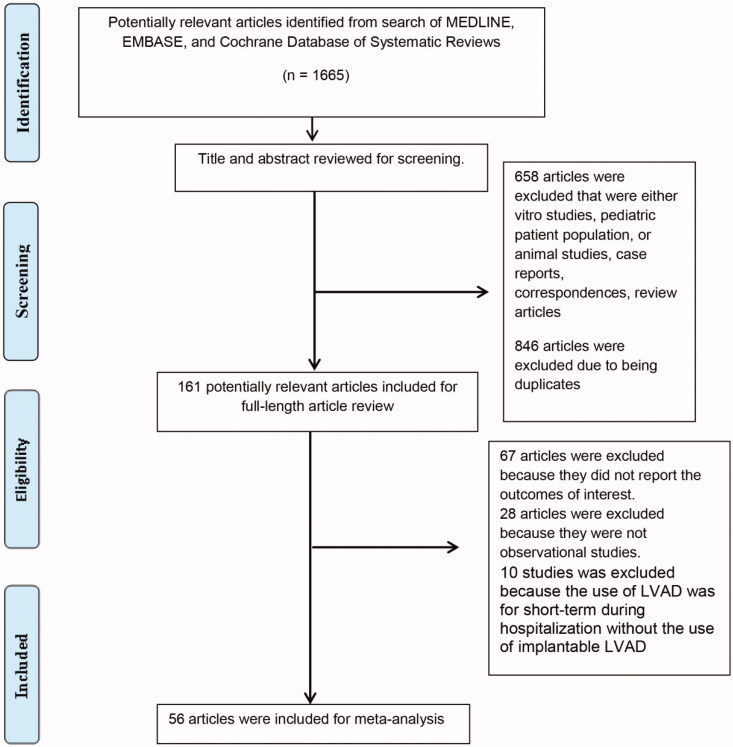
Flowchart depicting the systematic review of the literature.

Our search approach identified a total of 1,665 potentially eligible articles. We initially excluded 846 articles because they were case reports, correspondences, review articles, or studies involving in-vitro, animal, or pediatric patients. Six hundred fifty-eight duplicated articles were additionally excluded. After the review of 161 full-length articles, we subsequently excluded 67 articles because the data on AKI incidence and its associated mortality was not available, 28 articles because they were not observational studies or clinical trials, and 10 articles because they investigated AKI in short-term LVAD use, not implantable LVAD [79–88]. Therefore, 56 cohort studies [15–70] with a total of 63,663 adult patients were included in this meta-analysis. Figure 1 demonstrates by flowchart the systematic review of the literature. Table 1 shows the characteristics of the included studies. The kappa for systematic searches, selection of studies and data extraction were 1.00, 0.91 and 0.98, respectively

**Table 1.** Main characteristic of the included studies assessing the incidence of acute kidney injury in LVAD patients.

Study	Year	Country	Patients	Number	LVAD type	AKI definition	AKI and/or RRT incidence
Kaltenmaier et al. [40]	2000	Germany	Patient underwent LVAD implantation during 1988–1995 Mean age: 41.7 F: 22% Bridge to transplant: 71.8%	227	Pulsatile Berlin Heart System HeartMate 2000, Novacor	RRT	RRT 55/227 (24.2%)
Frazier et al. [35]	2001	USA	Cardiac transplant candidate underwent LVAD implantation Median age: 55 F: 17.1% Bridge to transplant: 100%	280	Pulsatile HeartMate Vented Electric LVAS (VE LVAS)	SCr ≥ 2.2 mg/dL or BUN value ≥50 mg/dL	AKI 158/280 (56.4%)
Haddad et al. [37]	2004	Canada	Patients underwent LVAS as a bridge to cardiac transplant during 1991–2003 Mean age: 43.8 F 31.4%	54	Pulsatile Thoratec VAD, Novacor	RRT	RRT 4/54 (7.4%)
Deng et al. [32]	2005	USA	Patients underwent LVAD implantation during 2002–2004 from Mechanical Circulatory Support Device (MCSD) database LVAD only: 82.7% LVAD + RVAD : 15.3% Bridge to transplant : 78.3% Destination therapy : 11.9%%	655	Continuous and Pulsatile (90%)	Not specified	AKI 85/655 (13%)
Topkara et al. [65]	2005	USA	Patients underwent LVAD implantation during 1996–2004	201	Pulsatile Thoratec HeartMate	RRT	RRT 65/201 (32.3%)
Feller et al. [34]	2007	USA	Patients underwent LVAD implantation during 2002–2005 Mean age: 54.1 F: 26% Bridge to transplant: 77.8% Destination therapy: 22.2% ICM: 48.1%	27	Continuous (51.9%) and Pulsatile (48.1%) Jarvik 2000, HeartMate XVE, Novacor	RRT	RRT 6/27 (22.2%)
Miller et al. [43]	2007	USA	Patient with end-stage heart failure on a waiting list for heart transplant underwent LVAD from 2005 to 2006 Mean age : 50.1 F: 21% ICM: 49/133 (37%)	133	Continuous HeartMate II	RRT	RRT 18/133 (13.5%)
Sandner et al. [52]	2008	Austria	Patients with end-stage heart failure underwent LVAD implantation as bridge to transplant during 1994–2007	92	Continuous (68.5%) and Pulsatile (31.5%) MicroMed DeBakey, HeartWare, Terumo DuraHeart	RRT	RRT : Total 33/92 (35.9%) Continuous LVAD 24/63 (38.1%) Pulsatile LVAD 9/29 (31.0%)
Pagani et al. [48]	2009	USA	Patient with end-stage heart failure on a waiting list for heart transplant underwent LVAD from 2005 to 2008 Bridge to transplant :100% Age: 50 F: 24% ICM: 43%	281	Continuous HeartMate II	RRT	RRT 30/281 (10.7%)
Alba et al. [21]	2009	Canada	Patients with end-stage heart failure underwent LVAD during 2001–2007 Mean age: 46 F 26.4% CKD: 15/53 (28.3%)	53	Continuous and Pulsatile Abiomed BVS500, Thoratec, Novacor, VE HeartMate, HeartMate II, Novacor	RIFLE criteria	AKI 24/53 (45.28%) RRT 15/53 (28.3%)
Slaughter et al. [59]	2009	USA	Patients with end-stage heart failure underwent LVAD implantation Mean age: 62.3 F: 15.5% ICM: 66.5%	200	Continuous (67%) and Pulsatile (33%) HeartMate II, H HeartMate XVE	RRT	RRT 35/200 (17.5%)
Genovese et al. [15]	2010	USA	Patients underwent LAVD or bi-VAD from 1996 to 2008 Age: 49.5 F: 20.2% LAVD:72% BiVAD: 28% Destination therapy: 6.1% Bridge to transplant: 82.8% Recovery support: 6.7% Postcardiotomy failure: 4.3%	163	Continuous (18%), pulsatile (54%), BiVAD (27.6%)	INTERMACS criteria: RRT or an increase in SCr ≥3 times baseline or SCr ≥ 5 mg/dL sustained for over 48 hours	AKI 22/163 (13.5%)
Demirozu et al. [31]	2011	USA	Patients underwent LVAD implantation during 2003–2009 Bridge to transplant 100%	107	Continuous HeartMate II	RRT	RRT 15/107 (14.0%) New RRT after LVAD 10/102 (9.8%)
John et al. [39]	2011	USA	Patients underwent HeartMate II implantation as a bridge to transplant after FDA approval during 2008–2010 from the INTERMACS registry Age 40–59: 53% F: 23% IABP: 33%	1496	Continuous HeartMate II	INTERMACS criteria: RRT or an increase in SCr ≥3 times baseline or SCr ≥ 5 mg/dL sustained for over 48 hours	AKI 129/1496 (8.6%)
Starling et al. [60]	2011	USA	Patient underwent LVAD implantation as a bridge to transplant in 2008 F: 22% IABP : 10% Inotrope support : 80%	338	169 Continuous HeartMateII 169 Pulsatile HeartMate XVE, Implantable VAD	INTERMACS criteria: RRT or an increase in SCr ≥3 times baseline or SCr ≥ 5 mg/dL sustained for over 48 hours	HM II AKI 17/169 (10.1%) HeartMate XVE, Implantable VAD AKI 21/169 (12.4%)
Strueber et al. [62]	2011	Germany, Australia, United Kingdom, USA, Austria	Patients with end-stage heart failure underwent LVAD implantation as a bridge to transplant during 2006–2008 Mean age: 48.5 F: 14% ICM: 40% Inotrope support: 100% IABP: 8%	50	Continuous HeartWare	INTERMACS criteria: RRT or an increase in SCr ≥3 times baseline or SCr ≥ 5 mg/dL sustained for over 48 hours	AKI : 5/50 (10%)
Park et al. [49]	2012	USA	Patient underwent LVAD implantation during 2005–2009 Destination therapy: 100%	414	Continuous HeartMate II	RRT	RRT 51/414 (12.3%)
Aaronson et al. [16]	2012	USA	Patient with end-stage heart failure underwent LVAD bridging to heart transplant during 2008–2010 Mean age: 52.4 F: 24.9%	140	Continuous HeartWare	INTERMACS criteria: RRT or an increase in SCr ≥3 times baseline or SCr ≥ 5 mg/dL sustained for over 48 hours	AKI 12/140 (8.6%)
Arnaoutakis et al. [23]	2012	USA	Patients underwent LVAD bridging to orthotropic heart transplant during 2005–2010 from UNOS database Mean age: 52 F: 18.1% Idiopathic cardiomyopathy: 50.7% Ischemic: 41.2%	1312	Continuous HeartMate II	RRT	RRT 106/1312 (8.1%)
Hasin et al. [38]	2012	USA	Patients underwent LVAD from 2007 to 2010 Mean age: 63 F: 19% CKD : 54% mean GFRs : 40 ml/min/1.73m² ischemic: 55% Bridge to transplant: 32 % Destination therapy: 68 %	83	Continuous HeartMate II	RRT	RRT 8/83 (9.6%)
Popov et al. [50]	2012	United Kingdom	Patient with end-stage heart failure underwent LVAD implantation during 2007–2011 Mean age: 51 F 15% ICM: 23.5% Preop IABP: 2.9%	34	Continuous HeartWare	RRT	RRT 12/34 (35.3%)
Yuan et al. [69]	2012	USA	Patients with end-stage heart failure underwent LVAD implantation during 2000–2012 Mean age: 50 F: 24.2% ICM : 29.1% Idiopathic: 46.3% Bridge to transplant: 53.9% destination therapy: 25.3%	182	Continuous and Pulsatile HeartMate XVE, HeartMate II	RRT	RRT 32/182 (17.6%)
Aissaoui et al. [20]	2013	Germany	Patients underwent LVAD implantation during 2001–2011 LAVD + RVAD: 45/488 (9.2%)	488	Continuous and Pulsatile Heartmate XVE, HeartMate II, HeartWare, VentrAssist, DuraHeart, Novacor, CorAide, Lionheart, Incor, DeBakey	Not specified	AKI : 199/488 (40.8%) LVAD alone 166/443 (37.5%) LVAD + RVAD 33/45 (73.3%)
Borgi et al. [26]	2013	USA	Patients with end-stage heart failure underwent LVAD during 2006–2011 Mean age: 52.8 F: 27% Chronic renal failure: 30 % Bridge to transplant: 68% ICM: 34% NICM: 66%	100	Continuous HeartMate II, HeartWare	RIFLE criteria	AKI 28/100 (28%) RRT 9/100 (9%)
Lok et al. [42]	2013	The Netherlands	End-stage heart failure patients who underwent LVAD placement as a bridge to transplantation during 2006–2011 Mean age: 45 F: 27% NICM: 71% ICM: 28%	85	Continuous HeartMate II	RRT	RRT: 9/85 (10.6%)
Ono et al. [47]	2012	USA	Patient underwent LVAD implantation	15	Continuous HeartMate II	RIFLE criteria	AKI 1/15 (6.7%)
Slaughter et al. [58]	2013	USA	Patients underwent LVAD implantation as a bridge to transplant in 2008	332	Continuous HeartWare	INTERMACS criteria: RRT or an increase in SCr ≥3 times baseline or SCr ≥ 5 mg/dL sustained for over 48 hours	AKI: 32/332 (9.6%)
Tsiouris et al. [66]	2013	USA	Patients underwent LVAD implantation during 2006–2011 Mean age: 52.5 F: 28.4% ICM: 29.5% NICM: 70.45% Bridge to transplant: 68.2%	88	Continuous HeartMate II, HeartWare	Not specified	AKI 22/88 (25%) RRT 6/88 (6.8%)
Brisco et al. [28]	2014	USA	Patients underwent LVAD or LVAD + RVAD implantation from INTERMACS database during 2006–2011 Mean Age: 54.5 F: 21.7% Bridge to transplant 39.3% Destination therapy 17.7% LVAD alone 92.1%	3363	Continuous (79.3%) and Pulsatile (20.7%)	Decrease in eGFR ≥ 25%	AKI: 336/3363 (10%)
Strueber et al. [61]	2014	Europe and Australia	Patients underwent LVAD implantation from ReVOLVE registry from 2009 to 2012	254	Continuous HeartWare	INTERMACS criteria: RRT or an increase in SCr ≥3 times baseline or SCr ≥ 5 mg/dL sustained for over 48 hours	10/254 (3.9%)
Naik et al. [46]	2014	USA	Patients underwent LVAD during 2008–2012 Bridge to transplant : 47.78% Destination therapy : 51.59%	157	Pulsatile (3.2%) HeartMate XVE Continuous Heartmate II, Heartware,	RIFLE criteria or AKIN criteria	RIFLE AKI 44/157 (28.02%) AKIN AKI 67/157 (42.7%) RRT 11/157 (7.0%)
Sumida et al. [63]	2014	Japan	Patients underwent LVAD implantation during 2011–2013 Mean age: 41.5 F: 19.3% ICM : 15.2% DCM: 64.5%	31	Not specified	KDIGO criteria	AKI 17/31 (54.8%) RRT 6/31 (19.4%)
Schechter et al. [53]	2014	USA	Patients underwent LVAD implantation from 2003 to 2012 Primary implantation: 50% Replacement: 50%	60	Not specified	Doubling of SCr	AKI All: 12/60 (20%) Primary implantation: 3/30 (10%) Replacement: 9/30 (30%)
Go et al. [36]	2015	USA	Patients underwent LVAD implantation during 2006–2014 Mean age: 54.3 F: 24% Chronic renal insufficiency: 40.5 Bridge to transplant: 49.5% Definitive treatment : 50.5%	200	Continuous HeartMate II, HeartWare	RIFLE criteria	AKI 74/200 (37.5%)
Topkara et al. [64]	2015	USA	Patients underwent LVAD implantation during 2004–2015 Mean age : 60.3 F: 20.1% ICM: 45.8% Bridge to transplant: 64% IABP : 27.5%	389	Continuous HeartMate II, HeartWare	RRT	Any RRT 44/389 (11.3%) New RRT after LVAD 38/383 (9.9%)
Deschka et al. [33]	2016	Germany	LVAD recipients with pre-operative biventricular impairment who received an additionally RVAD after a failed weaning attempt from cardiopulmonary bypass due to acute RV failure Age: 55.4 F: 20% ICM: 56% DCM: 40% Destination therapy: 36% Bridge to transplant: 64%	25	Not specified	RRT	RRT 9/25 (36%)
Nadziakiewicz et al. [45]	2016	Poland	Patients with end-stage heart failure underwent LVAD implantation during 2007–2014 ICM : 38.6%	44	Pulsatile (54.5%) Polvad MEV Continuous Heartware, HeartMate II	RRT	RRT 7/44 (15.9%)
Raichlin et al. [51]	2016	USA	End-stage heart failure Patients with preexisting renal dysfunction underwent LVAD implantation during 2009–2014 Age: 55.6 F: 19% Bridge to transplant: 50% ICM: 51% Mean Baseline GFR: 64.1	165	Continuous HeartMate II	RRT	RRT : 15/165 (9.1%)
Shehab et al. [55]	2016	Australia	Patients with dilated cardiomyopathy and severe biventricular failure who underwent dual HVAD implantation as a bridge to transplant during 2011–2014. Mean age: 45.6 F: 23%	13	Continuous HeartWare	INTERMACS criteria: RRT or an increase in SCr ≥3 times baseline or SCr ≥ 5 mg/dL sustained for over 48 hours	AKI 4/13 (30.7%)
Abbas et al. [17]	2017	USA	Patients underwent LVAD from the National Inpatient Sample (NIS) 2009–2011 database Mean age : 56 F :22.2% Chronic renal failure: 38%	4869	Not specified	ICD-9 codes and procedure code	AKI 1985/4869 (40.8%) RRT 277/4869 (5.7%)
Verma et al. [67]	2017	USA	Patient with end-stage heart failure underwent LVAD placement during 2010–2013 Mean age: 57.8 F: 23.7% CKD: 43.8% Bridge to transplant: 23.67%	169	Continuous	Increase in SCr of 0.3 mg/dL in 48 hours or 1.5 times from baseline in the seven days, or the need for RRT.	AKI 70/169 (47.3%) RRT 6/169 (3.5%)
Anjum et al. [22]	2018	USA	Patients underwent LVAD during 2003–2016 Mean age: 54.7 F 21.9% Bridge to transplant: 53.3%	520	Continuous HeartMate II (76.5%), HeartWare (23.5%)	RIFLE criteria	AKI 75/520 (14.4%)
Briasoulis et al. [27]	2018	USA	Patients underwent LVAD during 2009–2014 from the National Inpatient Sample (NIS) database Mean age: 55.42 F: 23.63% Chronic renal failure: 37.79%	3572	Continuous	ICD9, procedure codes	RRT 228/3572 (6.4%)
Catino et al. [29]	2018	USA	Patients with chronic heart failure underwent LVAD implantation during 2008–2014 Mean age: 52.8 F: 21% Bridge to transplant: 76.5% ICM: 40.7%	81	Continuous HeartMate II, HeartWare, Jarvik, Levacor	INTERMACS criteria: RRT or an increase in SCr ≥3 times baseline or SCr ≥ 5 mg/dL sustained for over 48 hours	AKI: 9/81 (11.1%) RRT 3/81 (3.7%)
Critsinelis et al. [30]	2018	USA	Patients underwent LVAD implantation during 2004–2016 Mean age: 54.7 F: 21.9% ICM: 45.6%	524	Continuous HeartMate II (77%) HeartWare (24%)	RIFLE criteria	AKI 75/524 (14.3%)
Kurihara et al. [41]	2018	USA	Patients underwent LVAD implantation during 2003–2016 Mean Age : 54.7 F: 21.9% CM : 45.4% Bridge to transplant: 283/526 (52.6%)	526	Continuous HeartMate II (76.6%), HeartWare (23.4%)	RIFLE criteria	AKI 75/526 (14.3%)
Muslem et al. [44]	2018	The Netherlands, USA	Patients underwent LVAD implantation during 2004–2015 Mean age: 52.4 F: 24% ICM: 34.4% Bridge to transplant: 155/241 (64.32%)	241	Continuous HeartMate II (90.9%), HeartWare (8.1%)	KDIGO criteria	AKI 169/241 (70.1%) RRT 23/241 (9.5%)
Schmack et al. [54]	2018	Germany	Symptomatic end-stage heart failure patients underwent LVAD from 2010 to 2017	68	Continuous HeartWare	RRT	RRT 32/68 (47.1%)
Shehab et al. [56]	2018	Australia	Patients underwent VAD implantation as a bridge to transplant from 2007 to 2016 Bridge to transplant: 100%	112	Continuous HeartWare	RRT	RRT All: 19/112 (17.0%)
Baxter et al. [25]	2019	USA	Patients underwent LVAD during 2008–2016 Mean age: 58.7 F: 17.3%	202	Continuous HeartMate II (90%), HeartMate III (3.3%), HeartWare (6.0%)	KDIGO criteria	AKI stage 2 and 3 66/202 (32.7%)
Silver et al. [57]	2019	USA	Patients with end-stage heart failure who were ineligible for transplantation underwent LVAD implantation during 2008–2013 from the national inpatient sample database	8362	Not specified	ICD-9 codes	AKI 4186/8362 (50.1%) RRT 426/8362 (5.1%)
Walther et al. [68]	2019	USA	Patient with end-stage heart failure underwent LVAD implantation during 2006–2015 from the national inpatient sample database	24140	Not specified	ICD-9-CM, procedure codes	AKI 13534/24140 (56.1%) RRT 1568/24140 (6.5%)
Zhigalov et al. [70]	2019	Germany	Patient underwent LVAD implantation from 2007 to 2018 Age: 63.5 F: 17.7% ICM: 56.5% Destination therapy: 55.6% Bridge to transplant: 7.3% Bright to candidacy: 5.6% Rescue therapy: 31.5%	124	Continuous HeartMate II (60%), HeartMate III (27%), HeartWare (13%)	RRT	RRT 35/124 (28.2%)
Adegbala et al. [18]	2019	USA	Patients underwent LVAD implantation from 2012 to 2014 from National readmission database Mean age: 60 F: 24%	3957	Not specified	RRT	RRT 178/3957 (4.5%)
Asleh et al. [24]	2019	USA	Patients underwent LVAD implantation during 2007–2017 Mean age: 60.4 F: 21.2% ICM: 46.6% Destination therapy: 67.2%	354	Continuous HeartMate II (80%), HeartMate III (2%) HeartWare (18%)	RRT	RRT 54/354 (15.3%)
Ahmed et al. [19]	2020	USA	Patients underwent LVAD implantation during 2009–2014 from the National Inpatient Sample database Mean age: 56 F: 23%	3511	Not specified	ICD9 codes, procedure codes	AKI 1996/3511 (56.9%) RRT 226/3511 (6.4%)

*Abbreviations.* AKI: acute kidney injury; AKIN: Acute Kidney Injury Network; CKD: chronic kidney disease; F: female; ICD: International Classification of Diseases; ICM: ischemic cardiomyopathy; KDIGO: Kidney Disease: Improving Global Outcomes; LVAD: left ventricular assist device; RVAD: right ventricular assist device; RRT: renal replacement therapy; SCr: serum creatinine; USA: Unites States of America; RIFLE: Risk, Injury, Failure, Loss of kidney function, and End-stage kidney disease.

### Incidence of AKI in LVAD patients

**Figure 2.**
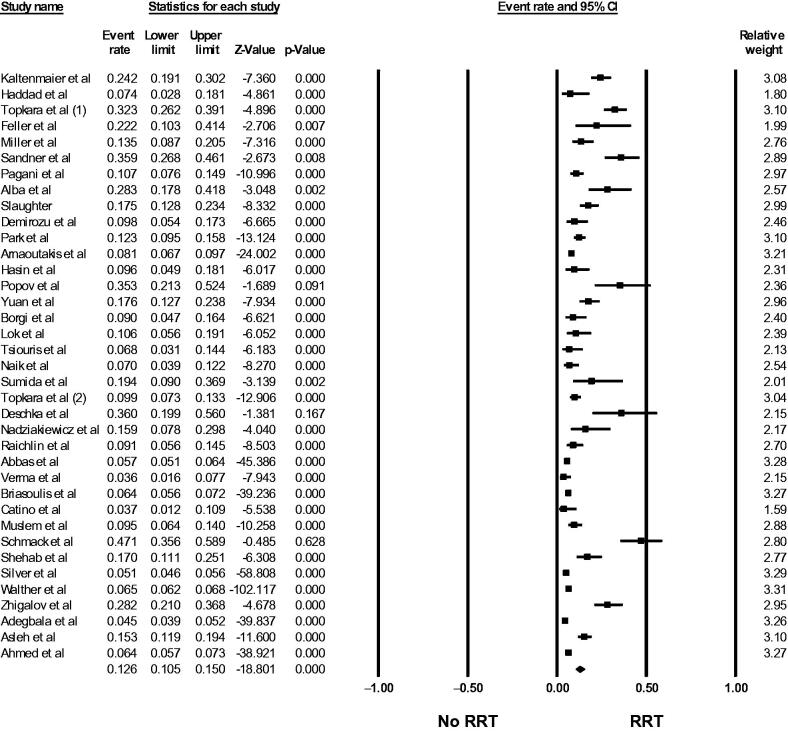
Forest plots of the included studies evaluating the incidence of severe AKI requiring RRT among LVAD patients. A diamond data marker represents the overall rate from the individual studies (square data marker) and 95% CI.

**Figure 3.**
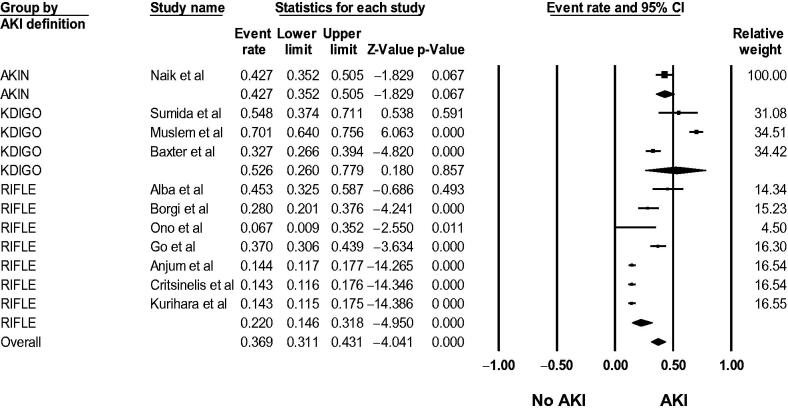
Forest plots of the included studies evaluating the incidence of AKI using standard AKI definitions (RIFLE, AKIN, and KDIGO criteria) among LVAD patients. A diamond data marker represents the overall rate from the individual studies (square data marker) and 95% CI.

Fifty-six studies [15–70] evaluated AKI incidence in LVAD patients. The pooled incidence of reported AKI was 24.9% (95%CI: 20.1%–30.4%, *I*^2^ = 99%, Supplementary Figure S1), and the pooled incidence of severe AKI requiring RRT was 12.6% (95%CI: 10.5%–15.0%, *I*^2^ = 95%, Figure 2). Using standard AKI definitions (RIFLE, AKIN, and KDIGO criteria), the pooled incidence of AKI was 36.9% (95%CI: 31.1%–43.1%, *I*^2^ = 97%, Figure 3).

**Figure 4.**
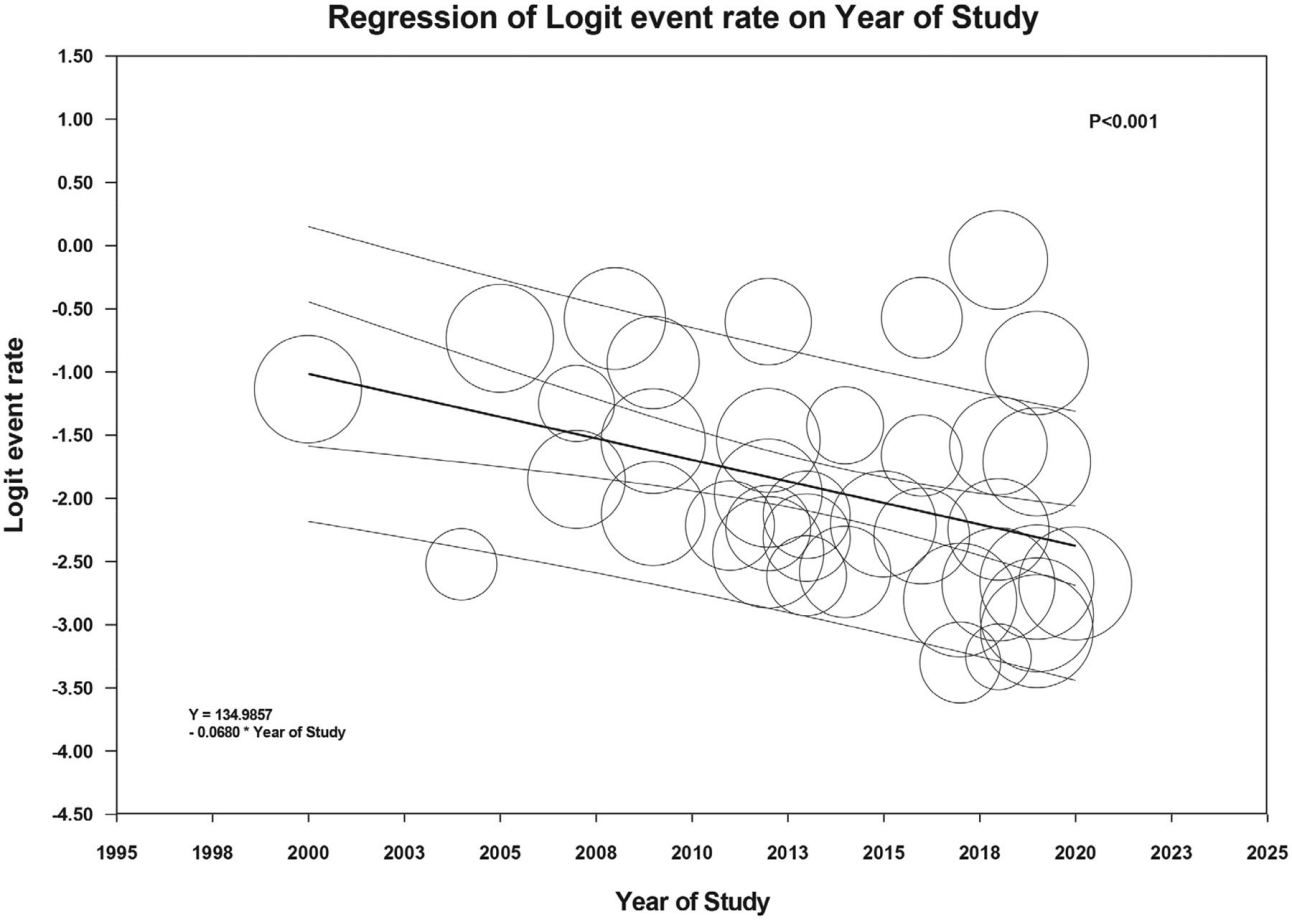
Meta-regression analysis demonstrated a significant negative correlation between the incidence of severe AKI requiring RRT and study year (slope = –0.068, *p* < .001)

AKI incidence did not differ significantly between types of LVAD (pulsatile vs. continuous flow) (*p* = .35) or indication of LVAD use (bridge to transplant vs. destination therapy) (*p* = 0.62). While meta-regression analysis did not demonstrate a significant association between study year and overall AKI incidence (*p* = .55) (Supplementary Figure S2), the study year was negatively correlated with the incidence of severe AKI requiring RRT (slope = −0.068, *p* < .001, Figure 4).

### Mortality associated with AKI in LVAD patients

Thirteen studies [18,21,22,24,26,40,44,46,52,54,57,63,65] evaluated mortality associated with AKI in LVAD patients, as shown in Table 2. The pooled odds ratio (OR) of 30-day mortality was 3.66 (95% CI, 2.00–6.70, *I*^2^ = 71%, Supplementary Figure S3) and the pooled OR of 1 year mortality was 2.22 (95% CI, 1.62–3.04, *I*^2^ = 0%, Supplementary Figure S4) in LVAD patients with AKI, compared with no AKI. The pooled OR of 30-day mortality was 7.52 (95% CI, 4.58–12.33, *I*^2^ = 73%, Supplementary Figure S5) and the pooled OR of 1-year mortality was 5.41 (95% CI, 3.63–8.06, *I*^2^ = 0%, Supplementary Figure S6) in LVAD patients with severe AKI requiring RRT, compared with no RRT.

**Table 2.** AKI associated Mortality in LVAD Patients.

Study	Year	Outcomes	Confounder adjustment	Quality assessment
Kaltenmaier et al. [40]	2000	30 days mortality RRT: 2.54 (1.36–4.74) 180 days mortality RRT: 8.54 (2.95–24.71)	None	Selection: 4 Comparability: 0 Outcome: 3
Topkara et al. [65]	2006	1-year mortality RRT: 5.10 (2.68–9.70)	None	Selection: 4 Comparability: 0 Outcome: 3
Sander et al. [52]	2008	Mortality RRT: 4.94 (1.98–12.36)	None	Selection: 4 Comparability: 0 Outcome: 3
Alba et al. [21]	2009	Mortality AKI: 14 (3.5–62) RRT: 12.18 (2.98–49.78) 15 days mortality AKI: 20 (2.32–172.29) 30 days mortality AKI: 15.95 (3.08–82.71) 90 days mortality AKI: 15.18 (3.84–59.99)	None	Selection: 4 Comparability: 0 Outcome: 3
Borgi et al. [26]	2013	AKI : 30 days mortality 33.94 (1.81–636.98) 180 days mortality 14.00 (2.75–71.26) 1 year mortality 4.59 (1.49–14.1) RRT : 30 days mortality RRT: 22.25 (3.10–159.70) 180 days mortality RRT:11.33 (2.40–53.60)	Chronic renal failure	Selection: 4 Comparability: 1 Outcome: 3
Naik et al. [46]	2014	AKI 30-day mortality AKI: 3.01 (1.15–7.91) 1-year mortality AKI: 1.85 (1.06–3.22)	For 30-day mortality: Age, black race, BMI, diabetes, bypass time, intraoperative PRBC use For 1-year mortality: BMI, diabetes, bypass time	Selection: 4 Comparability: 2 Outcome: 3
Sumida et al. [63]	2014	Mortality AKI: 5.42 (0.55–53.27) RRT: 23 (2.48–213.70)	None	Selection: 4 Comparability: 0 Outcome: 3
Anjum et al. [22]	2018	AKI : Mortality: 1.54 (1.02–2.32) In hospital mortality: 1.25 (0.68–2.33) 180 days mortality: 2.08 (1.20–3.60) 1 year mortality: 2.34 (1.41–3.89)	Age, Body mass index, body surface area, previous cardiac surgery, preoperative inotrope use, LVAD type, severe tricuspid regurgitation	Selection: 4 Comparability: 2 Outcome: 3
Muslem et al. [44]	2018	30 days mortality AKI: 1.82 (0.71–4.66) RRT: 3.71 (1.38–9.96) 1 year mortality AKI: 2.02 (1.02–3.99) RRT: 4.97 (2.03–12.13)	None	Selection: 4 Comparability: 0 Outcome: 3
Schmack et al. [54]	2018	30-day mortality 5.85 (1.63–20.8)	None	Selection: 4 Comparability: 0 Outcome:: 3
Adegbala et al. [18]	2019	In hospital mortality RRT : 9.57 (6.21–14.75)	None	Selection: 4 Comparability: 0 Outcome: 3
Silver et al. [57]	2019	In hospital mortality AKI: 4.63 (3.88–5.53) RRT: 10.66 (8.67–13.11)	None	Selection: 4 Comparability: 0 Outcome: 3
Asleh et al. [24]	2019	In-hospital mortality RRT: 10.21 (4.68–22.28) 1-year mortality RRT: 5.95 (3.20–11.05) Mortality RRT: 2.86 (1.90–4.33)	Age, sex, diabetes mellitus, redo sternotomy, destination LVAD, right atrial pressure/pulmonary capillary wedge pressure ratio	Selection: 4 Comparability: 2 Outcome: 3

*Abbreviations.* AKI: acute kidney injury; BMI: body mass index; LVAD: left ventricular assist device; PRBC: pack red blood cell; RRT: renal replacement therapy.

### Publication bias evaluation

Using funnel plots (Supplementary Figure S7–10) and Egger’s regression asymmetry tests, there was no significant publication bias found in this meta-analysis (*p*-values = .78, .25, .53, and .59, respectively).

## Discussion

This meta-analysis supports that AKI is a common complication after LVAD implantation. The pooled incidence of post-LVAD AKI (using standard AKI definitions) and severe AKI requiring RRT was 37% and 13%, respectively. We found no significant difference in AKI incidence after adjusting for LVAD indication (bridging vs. destination therapy). Moreover, our analysis did not show any difference in AKI incidence between pulsatile and continuous flow LVAD devices. It was also noted that the incidence of AKI was higher (37% vs. 25%) when using standard AKI criteria, such as RIFLE, AKIN, and KDIGO. This may indicate that defining AKI using consensus criteria may improve the sensitivity of detecting AKI in LVAD patients. This meta-analysis further identified that AKI incidence remained constant over time, while the need for RRT due to AKI decreased significantly in more recent studies.

The mechanisms of AKI among LVAD patients are complex and can be multifactorial [5,89,90]. Mechanical stress on red blood cells traveling through the LVAD leads to constant low-level hemolysis, potentially resulting in pigment nephropathy [5]. These patients also tend to have acquired von Willebrand disease, as the von Williebrand factor multimers suffer fragmentation when passing through the LVAD pump, leading to subsequent increased risk of AKI due to decreased effective blood volume secondary to bleeding from arteriovenous malformations or severe epistasis [5,8]. An additional concern is, the development of right heart failure following LVAD implantation, which is observed in approximately 20–50% of patients [14,91–94]. This right heart failure could further potentiate renal venous congestion, compromised net effective renal perfusion pressure and decrease GFR [95,96]. Hemodynamic instability in the immediate post-operative period could exacerbate kidney ischemia and lead to acute tubular necrosis. Accelerated thrombogenicity secondary to the LVAD pump and blood stasis may trigger renal microemboli, as evidenced by the presence of kidney infarctions [97]. Yet an another proposed hypothesis for the development of worsening kidney function in LVAD patients is that the continuous flow of the LVAD might lead to a proliferation of afferent arteriolar smooth muscle cells and periarteriolitis, which causes an eventual decline in eGFR [98]. However, in our study, the incidence of AKI was similar between pulsatile-flow and continuous-flow LVADs, suggesting that the lack of pulsatility from continuous-flow LVADs might not be the cause of associated AKI. On the other hand, currently only a limited amount of pulsatility can be generated by LVADs using periodic speed steps, and it is considerably smaller in both flow increase and rate than what is found with natural pulsatile circulation [99]. Given the ongoing efforts to advance LVAD technology, future studies are needed to evaluate whether or not improvements in pulsatile-flow LVADs can reduce the incidence of post-LVAD implantation AKI.

The findings from our study demonstrated that LVAD patients who developed AKI had greater odds of 30-day and 1-year mortality. The pooled odds ratios were even higher in patients with severe AKI requiring RRT. It is emphasized that even an occurrence of AKI following LVAD implantation has long-lasting negative clinical impacts, especially if dialysis is required [40, 100]. Post-implantation AKI is associated with right ventricular failure and arrhythmias, both of which are, in turn, associated with increased mortality [28]. Our study shows that LVAD patients with severe AKI requiring RRT are associated with 7.5-fold and 5.4-fold increased risks of 30-day and 1-year mortality, respectively. While the findings of our study suggested no significant changes in overall AKI incidence over the study years, the incidence of severe AKI requiring RRT appeared to decrease over study year significantly. This finding suggests potential improvements in the prevention, mitigation, and clinical management of severe AKI in LVAD patients. Interventions proposed to mitigate the incidence and severity of post-LVAD implantation AKI include maintenance of high mean arterial pressures (MAP) and coronary perfusion rates [96], inotropic support when needed, frequent monitoring of MAP *via* audible doppler ultrasound in combination with calibrated blood pressure measurement devices, and maintaining central venous pressures between 8 and 12 mm Hg *via* diuretics or extracorporeal ultrafiltration. Avoiding nephrotoxic medications postoperatively until hemodynamic stability is achieved has also been recommended. In patients with severe right heart failure, right ventricular assist devices may help decrease right-sided venous congestion and improve renal perfusion [96]. Future studies are required to assess whether these measures can significantly help to reduce AKI incidence or promote AKI recovery among LVAD patients, to improve patient survival rates ultimately.

Our systematic and meta-analysis is subject to certain limitations. First, all studies were observational in design, making them susceptible to potential selection bias. The potential sources of this heterogeneity included differences in variation in baseline characteristics (e.g., age, sex, ethnicity, and underlying chronic kidney disease), LVAD types, indications for LVAD, and outcome ascertainments. Second, the incidence of AKI is predisposed to several confounding factors. Our meta-analysis had a high degree of heterogeneities. However, we performed subgroup analyses after applying standardized AKI definitions and conducted meta-regression analyses assessing the effects of the study year, LVAD types (pulsatile vs. continuous flow), and indications for LVAD implantation (bridge to transplant vs. destination therapy) on AKI incidence. The results from these additional analyses provided clinical insights that may highlight and stimulate the need for additional research to intervene on AKI in LVAD patients. Third, the data on the use of peritoneal dialysis as a modality of RRT in LVAD patients is limited, and all of the included studies defined RRT as either non-peritoneal continuous or intermittent renal replacement therapies. Lastly, AKI diagnoses in the included studies were solely based on the change in serum creatinine, which might underestimate the incidence of AKI [101–105]. Data on urine output or other AKI biomarkers data were limited [104, 106] Furthermore, future studies using artificial intelligence to predict AKI among LVAD patients are needed [107].

In conclusion, AKI is a common complication among LVAD patients. There have been some potential improvements in the incidence rates of severe AKI requiring RRT in LVAD patients. AKI, while on LVAD, is associated with increased 30-day and 1-year mortality.

## Supplementary Material

Supplemental Material

Supplemental Material
