# Supplementary material for: Incidence and impact of acute kidney injury on patients with implantable left ventricular assist devices: a Meta-analysis
Source: Ren Fail. 2020 May 20;42(1):495–512. doi: 10.1080/0886022X.2020.1768116 (PMC7301695; doi:10.1080/0886022X.2020.1768116)

Online Supplementary Data

Search Strategy

Database: OVID MEDLINE

1. ventricular assist device.mp
2. exp ventricular assist device/
3. LVAD.mp
4. VAD.mp
5. 1 or 2 or 3 or 4
6. exp acute kidney injury/
7. acute kidney injury$.mp
8. exp acute renal failure/
9. acute renal failure$.mp.
10. exp renal insufficiency/
11. renal insufficiency$.mp.
12. exp dialysis/
13. dialysis$.mp.
14. hemodialysis$.mp.
15. renal replacement therapy$.mp.
16. hemofiltration$.mp.
17. hemodiafiltration$.mp.
18. 6 or 7 or 8 or 9 or 10 or 11 or 12 or 13 or 14 or 15 or 16 or 17
19. 5 and 18

Database: EMBASE

('left ventricular assist device' OR 'lvad' OR 'ventricular assist device') AND ('acute kidney failure' OR 'acute kidney injury' OR 'renal replacement therapy' OR dialysis)

Database: Cochrane Database of Systematic Reviews

('left ventricular assist device' OR 'lvad' OR 'ventricular assist device') AND ('acute kidney failure' OR 'acute kidney injury' OR 'renal replacement therapy' OR dialysis)

Figure S1. Forest plots of the included studies evaluating overall incidence of AKI among patients on LVAD. A diamond data marker represents the overall rate from individual study (square data marker) and 95% CI.

Study name	Event rate	Lower limit	Upper limit	Z-Value	p-Value	Relative weight
Frazier et al	0.564	0.506	0.621	2.145	0.032	3.49
Deng et al	0.130	0.106	0.158	-16.367	0.000	3.50
Alba et al	0.453	0.325	0.587	-0.686	0.493	3.15
Genovese et al	0.135	0.091	0.196	-8.104	0.000	3.27
John et al	0.086	0.073	0.102	-25.629	0.000	3.53
Starling et al	0.101	0.063	0.156	-8.566	0.000	3.21
Strueber et al (1)	0.100	0.042	0.219	-4.661	0.000	2.56
Aaronson et al	0.086	0.049	0.145	-7.841	0.000	3.08
Aissaoui et al	0.375	0.331	0.421	-5.217	0.000	3.52
Borgi et al	0.280	0.201	0.376	-4.241	0.000	3.29
Ono et al	0.067	0.009	0.352	-2.550	0.011	1.22
Slaughter et al	0.096	0.069	0.133	-12.035	0.000	3.37
Tsiouris et al	0.250	0.171	0.351	-4.463	0.000	3.23
Brisco et al	0.100	0.090	0.111	-38.228	0.000	3.56
Strueber et al (2)	0.039	0.021	0.072	-9.901	0.000	3.02
Naik et al	0.427	0.352	0.505	-1.829	0.067	3.42
Sumida et al	0.548	0.374	0.711	0.538	0.591	2.90
Schechter et al	0.200	0.117	0.320	-4.295	0.000	3.02
Go et al	0.370	0.306	0.439	-3.634	0.000	3.45
Shehab et al	0.308	0.120	0.591	-1.349	0.177	2.17
Abbas et al	0.408	0.394	0.422	-12.809	0.000	3.58
Verma et al	0.414	0.342	0.490	-2.220	0.026	3.43
Anjum et al	0.144	0.117	0.177	-14.265	0.000	3.49
Catino et al	0.111	0.059	0.200	-5.882	0.000	2.92
Critsinelis et al	0.143	0.116	0.176	-14.346	0.000	3.49
Kurihara et al	0.143	0.115	0.175	-14.386	0.000	3.49
Muslem et al	0.701	0.640	0.756	6.063	0.000	3.46
Baxter et al	0.327	0.266	0.394	-4.820	0.000	3.44
Silver et al	0.501	0.490	0.511	0.109	0.913	3.58
Walther et al	0.561	0.554	0.567	18.799	0.000	3.58
Ahmed et al	0.568	0.552	0.585	8.092	0.000	3.58
	0.249	0.201	0.304	-7.854	0.000	

Event rate and 95%CI

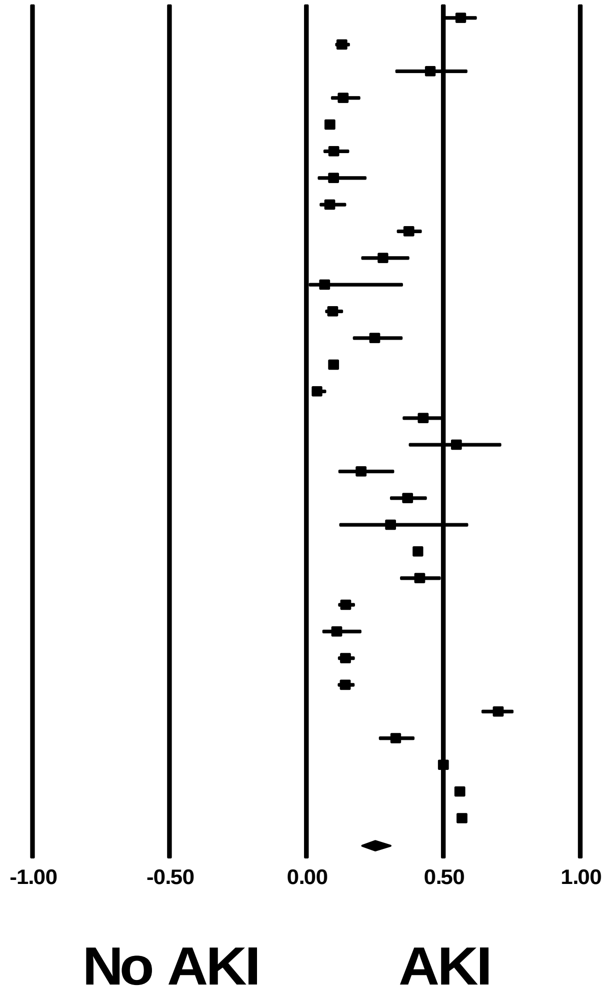

Figure S2. Meta-regression analysis demonstrated that the year of study did not significantly affect the overall incidence of AKI (*P*=0.55) among patients on LVAD.

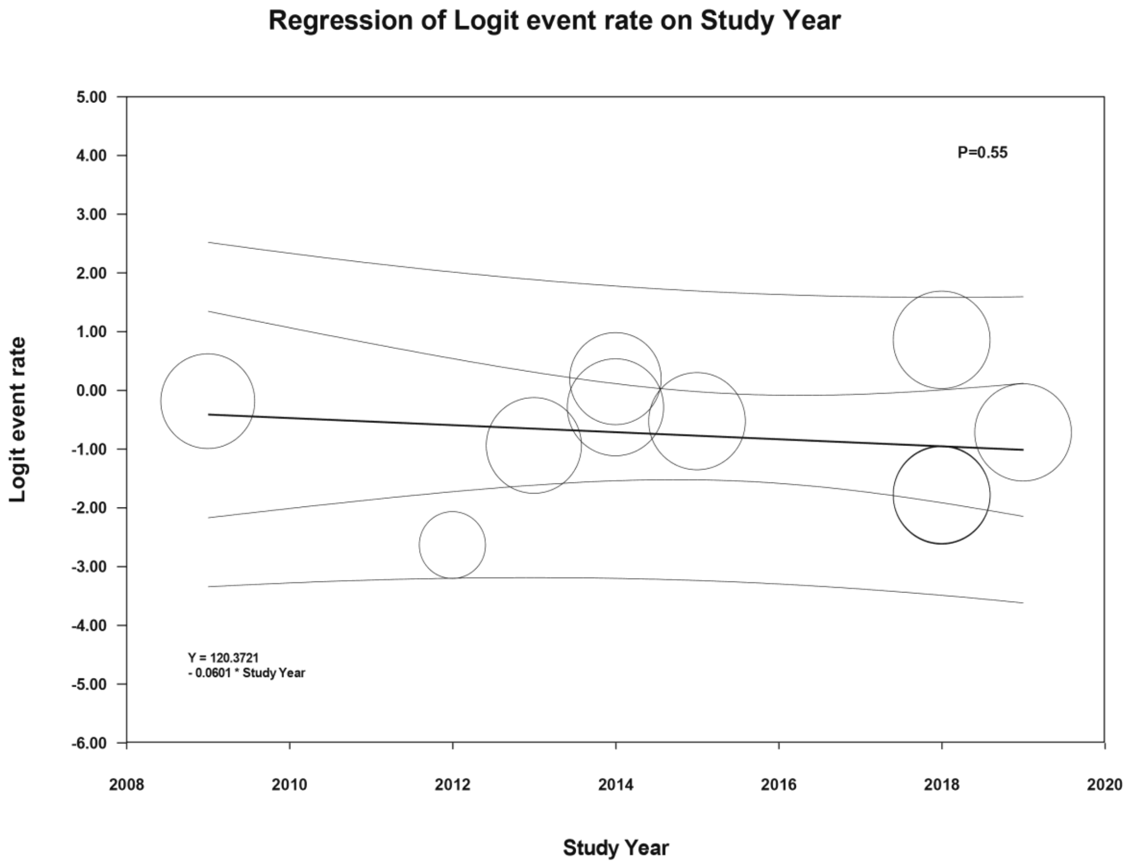

Figure S3. Forest plots of the included studies evaluating 30-day mortality among patients receiving LVAD with AKI. A diamond data marker represents the overall rate from individual study (square data marker) and 95% CI.

Study name	Statistics for each study					Relative weight
	Odds ratio	Lower limit	Upper limit	Z-Value	p-Value	
Alba et al	15.950	3.078	82.654	3.299	0.001	8.54
Anjum et al	1.250	0.675	2.314	0.710	0.478	18.74
Borgi et al	33.940	1.809	636.701	2.356	0.018	3.59
Muslem et al	1.820	0.710	4.663	1.248	0.212	14.88
Naiket al	3.010	1.148	7.894	2.240	0.025	14.61
Schmack et al	5.850	1.638	20.897	2.719	0.007	11.45
Silver et al	4.630	3.878	5.527	16.953	0.000	22.81
Sumida et al	5.420	0.551	53.341	1.449	0.147	5.37
	3.661	2.000	6.701	4.208	0.000	

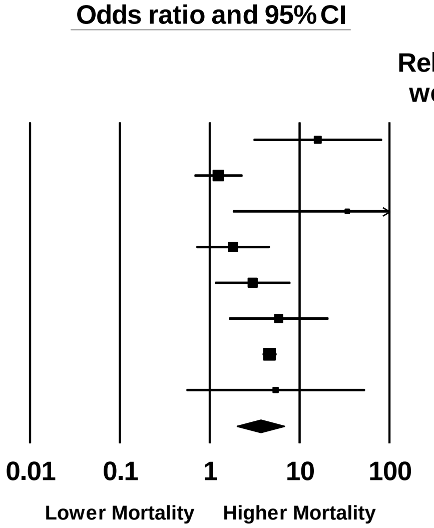

Figure S4. Forest plots of the included studies evaluating 1-year mortality among patients receiving LVAD with AKI. A diamond data marker represents the overall rate from individual study (square data marker) and 95% CI.

Study name	Statistics for each study					Relative weight
	Odds ratio	Lower limit	Upper limit	Z-Value	p-Value	
Borgi et al	4.590	1.492	14.120	2.658	0.008	7.87
Naik et al	1.850	1.061	3.224	2.170	0.030	32.19
Anjum et al	2.340	1.409	3.887	3.284	0.001	38.59
Muslem et al	2.020	1.021	3.995	2.021	0.043	21.36
	2.217	1.618	3.038	4.951	0.000	

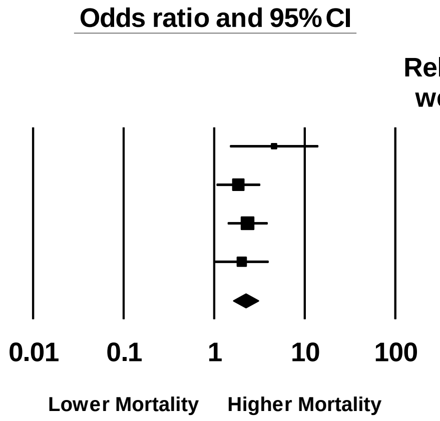

Figure S5. Forest plots of the included studies evaluating 30-day mortality among patients receiving LVAD with severe AKI on RRT. A diamond data marker represents the overall rate from individual study (square data marker) and 95% CI.

Study name	Statistics for each study					Relative weight
	Odds ratio	Lower limit	Upper limit	Z-Value	p-Value	
Adegbala et al	9.570	6.210	14.749	10.234	0.000	21.01
Asleh et al	10.210	4.679	22.277	5.837	0.000	15.43
Borgi et al	22.250	3.100	159.699	3.085	0.002	5.04
Kaltenmaier et al	2.540	1.361	4.742	2.927	0.003	17.90
Muslem et al	3.710	1.381	9.967	2.600	0.009	12.53
Silver et al	10.660	8.669	13.108	22.434	0.000	23.97
Sumida et al	23.000	2.478	213.503	2.758	0.006	4.12
	7.516	4.581	12.330	7.986	0.000	

Odds ratio and 95% CI

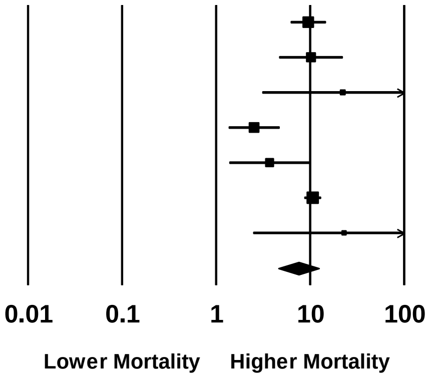

Figure S6. Forest plots of the included studies evaluating 1-year mortality among patients receiving LVAD with severe AKI on RRT. A diamond data marker represents the overall rate from individual study (square data marker) and 95% CI.

Study name	Statistics for each study					Odds ratio and 95% CI	
	Odds ratio	Lower limit	Upper limit	Z-Value	p-Value		Relative weight
Topkara et al	5.100	2.681	9.703	4.965	0.000		38.53
Muslem et al	4.970	2.033	12.149	3.516	0.000		19.95
Asleh et al	5.950	3.202	11.057	5.641	0.000		41.51
	5.409	3.629	8.063	8.287	0.000		

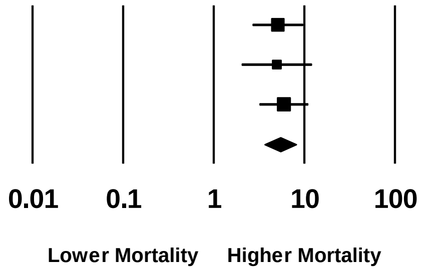

Figure S7. Funnel plot evaluating for publication bias evaluating 30-day mortality among patients on LVAD with AKI.

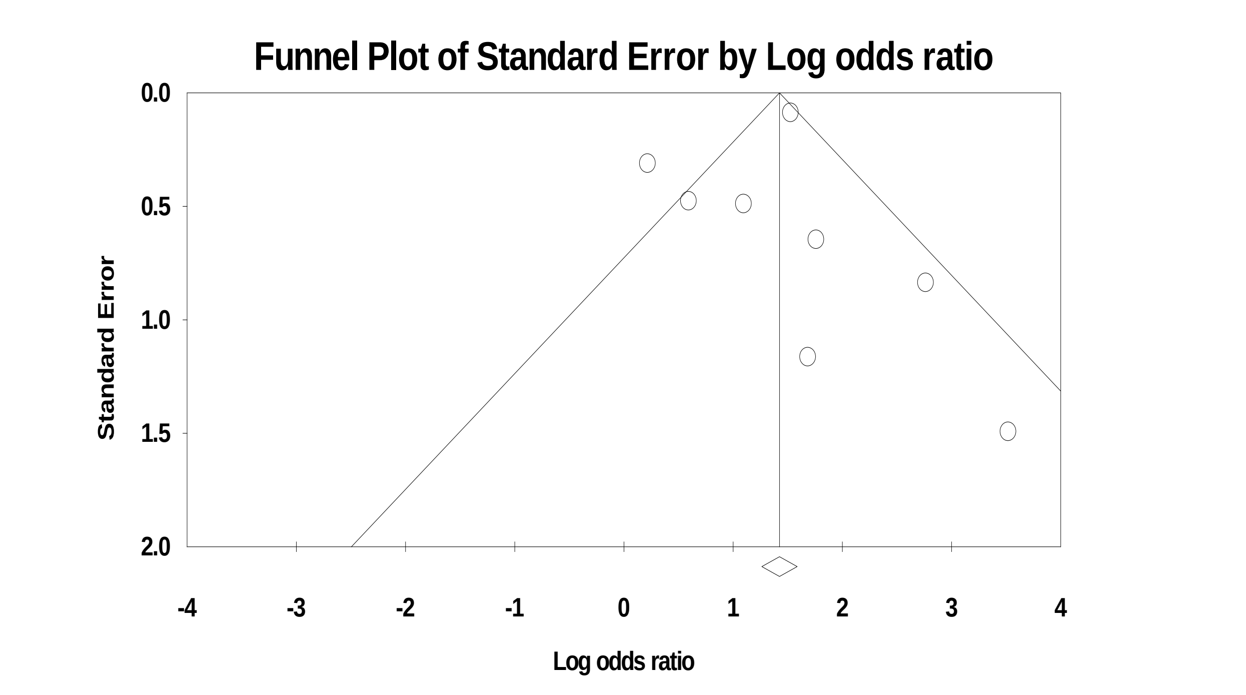

Figure S8. Funnel plot evaluating for publication bias evaluating 1-year mortality among patients on LVAD with AKI.

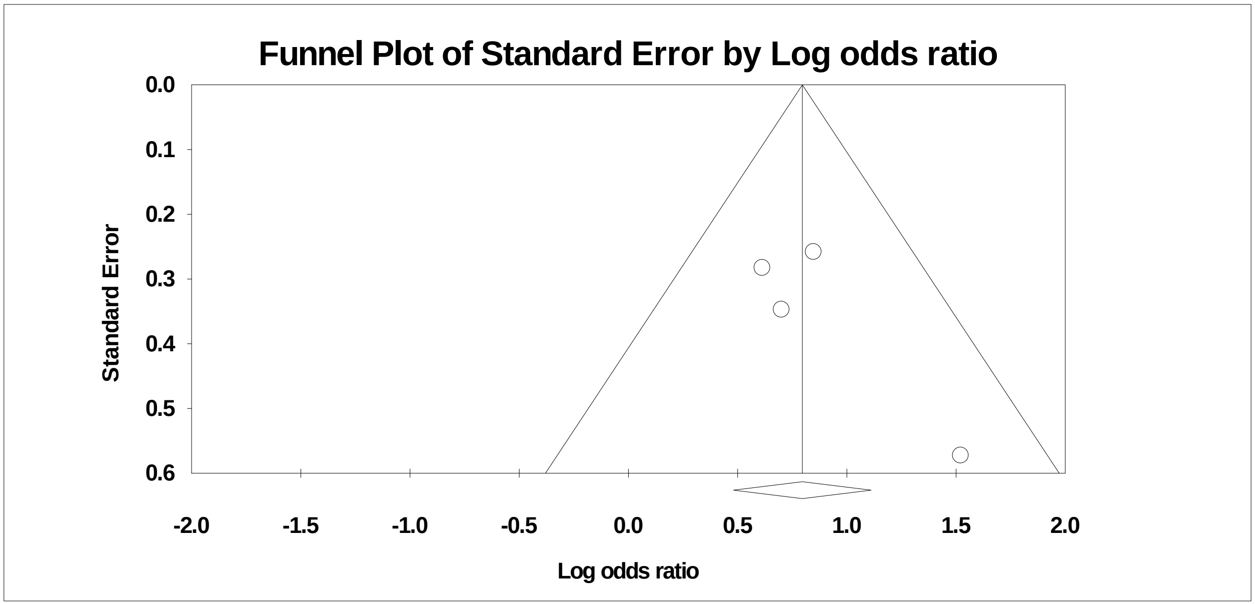

Figure S9. Funnel plot evaluating for publication bias evaluating 30-day mortality among patients on LVAD with severe AKI on RRT.

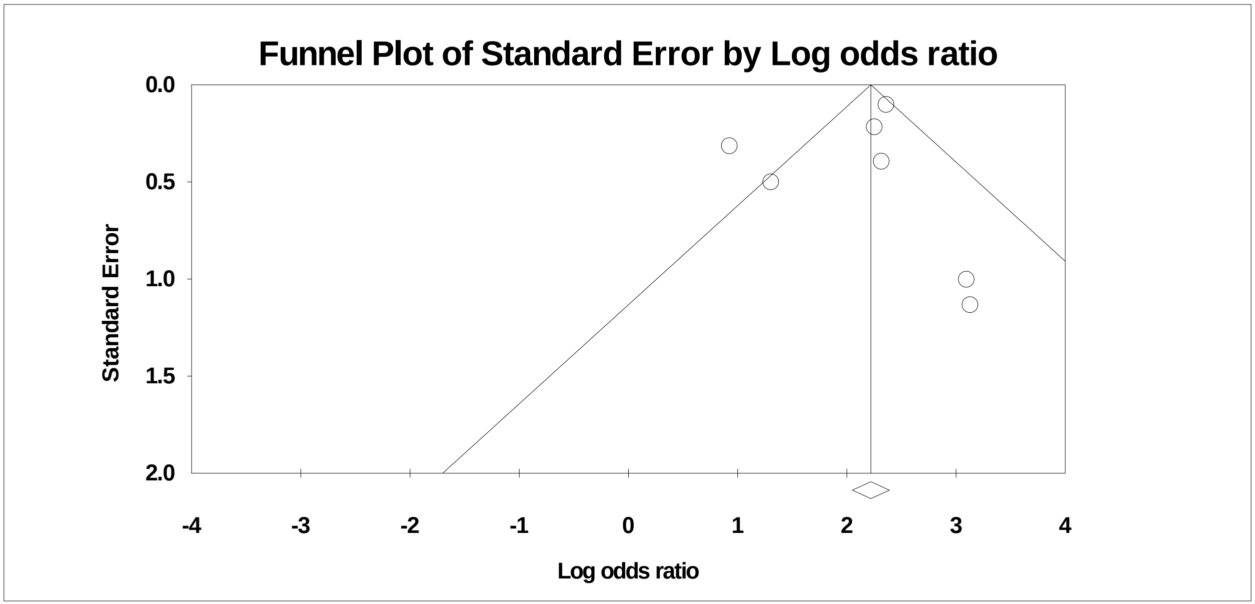

Figure S10. Funnel plot evaluating for publication bias evaluating 1-year mortality among patients on LVAD with severe AKI on RRT.

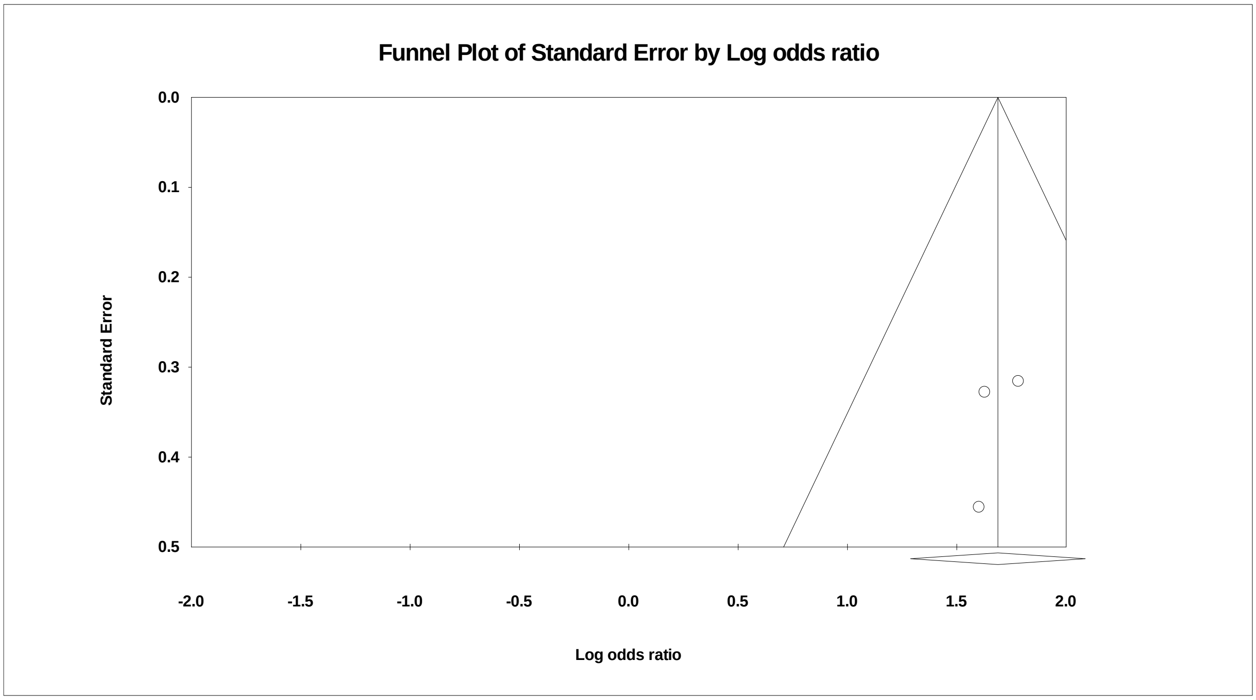

Supplement: Supplemental Material [file IRNF_A_1768116_SM0939.pdf]
